# Aeromonas caviae: A rare case of hepatic abscess

**DOI:** 10.3205/dgkh000612

**Published:** 2026-01-09

**Authors:** Aristos Aristodimou, Zacharias Raptopoulos, Andreas Kostis, Elena Xenofontos, Loukia Dramiotou

**Affiliations:** 1 Internal Medicine Department, Limassol General Hospital, State Health Organization Services, Limassol, Cyprus

**Keywords:** Aeromonas caviae, liver abscess, lung neoplasms, bacteremia, pancreatic neoplasms

## Abstract

**Aim:**

The aim of this report is to present, to our knowledge, the first case of *Aeromonas (A.) caviae* liver abscess.

**Methods:**

The following manuscript describes a case of a 75-year-old female patient with past medical history of pancreatic cancer, lung cancer, hypothyroidism, hypertension and chronic venous insufficiency who was diagnosed with *A. caviae* bacteremia and multiple liver abscesses in the right lobe. The patient was treated conservatively, with intravenous antibiotics for two months with adequate recovery.

**Discussion:**

*Aeromonas* spp. is a Gram-negative, facultative anaerobic microorganism that lives in aquatic environments. There are many subtypes of *Aeromonas* spp., with the commonest being *A. hydrophila*, *A. sobria* and *A. caviae*. They tend to cause gastrointestinal and hepatobiliary disease in immunocompromised people, mostly those with underlined disease or malignancy of the hepatobiliary tract.

**Conclusion:**

*Aeromonas* spp. has been identified in chlorinated tap water, worldwide. Global warming contributes not only to the reproduction of more bacteria, but also to the development of antibiotic resistance genes and biofilms. Besides the fact that liver abscess due to *Aeromonas caviae* is rare; the following report highlights a major, public health concern, that is Global warming, and the devastating impact it can have on healthcare.

## Zusammenfassung

**Ziel:**

Ziel dieses Berichts ist es, den unseres Wissens ersten Fall eines Leberabszesses durch *Aeromonas (A.) caviae* vorzustellen.

**Methoden:**

Das folgende Manuskript beschreibt den Fall einer 75-jährigen Patientin mit Bauchspeicheldrüsenkrebs, Lungenkrebs, Schilddrüsenunterfunktion, Bluthochdruck und chronischer Veneninsuffizienz in der Vorgeschichte. Bei ihr wurden eine *A. caviae*-Bakteriämie und mehrere Leberabszesse im rechten Leberlappen diagnostiziert. Die Patientin wurde zwei Monate lang konservativ mit intravenösen Antibiotika behandelt und erholte sich ausreichend.

**Diskussion:**

*Aeromonas* spp. ist ein Gramnegativer, fakultativ anaerober Mikroorganismus, der in Gewässern lebt. Es gibt viele Subtypen, wobei *A. hydrophila*, *A. sobria* und *A. caviae* die häufigsten sind. Sie neigen dazu, bei immungeschwächten Personen, meist bei Patienten mit einer bestehenden Erkrankung oder Malignität des Leber- und Gallentrakts, gastrointestinale und hepatobiliäre Erkrankungen zu verursachen.

**Schlussfolgerung:**

*Aeromonas* spp. wurde weltweit in gechlortem Leitungswasser nachgewiesen. Die globale Erwärmung trägt nicht nur zur Vermehrung von Bakterien bei, sondern auch zur Entwicklung von Antibiotikaresistenzgenen und Biofilmen. Neben der Tatsache, dass Leberabszesse durch *A. caviae* selten sind, beleuchtet der folgende Bericht ein großes Gesundheitsproblem: die globale Erwärmung und ihre verheerenden Auswirkungen auf die Gesundheitsversorgung.

## Introduction

Liver abscess is defined as a pus-filled mass in the liver, most commonly located in the right lobe (richer in blood supply), but also seen in the left lobe and caudate lobe [1]. The mechanism of abscess development includes direct hepatic injury, disseminated intra-abdominal infection (bowel fluid leaks into the portal circulation and reaches the liver) and biliary disease (stones, strictures, malignancy) [2], [3]. Most of them are either pyogenic or amebic, even though fungal and parasites can cause liver abscess as well [3]. Pyogenic abscesses are usually polymicrobial in origin with the most predominant isolates being *Escherichia coli*, *Klebsiella pneumoniae*, *Streptococcus* spp., *Staphylococcus* spp. and other anaerobic bacteria [3]. Amebic liver abscess is the commonest extra intestinal manifestation of human invasive amebiasis complicating 9% of intestinal disease [4]. 

*Aeromonas* spp. can rarely cause liver abscess with no reports of *A. caviae* as the causative agent so far [5]. *Aeromonas* is a Gram-negative facultative anaerobic organism that normally lives in aquatic environments, soil, fish, animals and foodstuff [6]. There are many subspecies with the commonest being *A. hydrophila*, *A. sobria* and *A. caviae* [6]. Infection causes gastrointestinal disease (gastroenteritis), hepatobiliary disease, soft tissue infection, septic shock and in rare cases, pleuropulmonary disease [7]. It can affect healthy individuals, but those at higher risk are patients with liver cirrhosis and malignancies [7]. Here in, we present a rare case of hepatic abscesses in a patient found to have *A. caviae* bacteremia. 

## Case description

The patient is a 75-year old woman with past medical history of pancreatic cancer (surgical resection – Whipple’s procedure), lung cancer (lobectomy of the left upper lobe), hypothyroidism, hypertension, aortic stenosis and chronic venous insufficiency who presented to the Accident and Emergency Department due to a 24-hour history of two episodes of vomiting along with three episodes of watery diarrhea. She also complained of fever and chills the past 24 hours. There was no recent use of antibiotics noted. 

Upon examination, the patient had temperature of 38.7°C and was hemodynamically stable (blood pressure 110/80 mmHg). Auscultation of the heart revealed a systolic murmur over the aortic valve region (known history of aortic stenosis). Abdomen was soft and non-tender with normal bowel sounds. Neither the spleen, nor the liver were palpable. Rest of the physical examination was normal. 

Blood tests were significant for leukocytosis (14,590 cells/µL), associated with neutrophilia (14,050 cells/µL), microcytic anemia (Hb11.8 g/dL with mean corpuscular volume of 90.2 fL) and elevated inflammatory markers (erythrocyte sedimentation rate 34 mm/hr, C-reactive protein (CRP) 127mg/L and procalcitonin levels >10 ng/mL). Liver enzymes were also elevated with AST 62 U/L and ALT 42 U/L. Blood cultures were obtained and later returned positive for *A. caviae*, sensitive only to trimethoprim/sulfamethoxazole (TMP/SMX). Stool culture was negative for *Clostridium difficile* and common microorganisms (salmonella and shigella spp.). COVID-19 rapid test was also negative. The derangement noted in the liver function tests prompted to requesting an abdominal ultrasound with evidence of two hypoechoic lesions in the right lobe of the liver (5.2 cm x 2.5 cm and 6.8 cm x 4 cm). A computed-tomography (CT) scan of the abdomen and pelvis followed that revealed two hypoechoic liver lesions in the right liver lobe with irregular borders, measuring 4.1 cm x 3.5 cm and 6.7 cm x 3.8 cm – resembling hepatic abscesses. The patient was evaluated by the surgical team who suggested conservative treatment with intravenous antibiotics. A follow-up CT scan of the abdomen and pelvis was done two weeks later and revealed significant reduction in the size of both lesions described above (2.4 cm x 3.5 cm and 3.3 cm x 3.0 cm). She received one month of targeted, intravenous, antibiotic therapy (with TMP/SMX) and her blood tests, upon discharge, showed significant improvement (white cell count of 5,250 cells per µL, CRP 1g/dL). The patient was discharged with one month of oral antibiotics (TMP/SMX), and a follow-up appointment with the surgical team and the infectious diseases consultant. Imaging studies (CT abdomen – pelvis) were repeated, upon completion of oral antibiotic treatment, and showed further reduction in the size of the abscesses (1.5 cm x 0.9 cm and 2.7 cm x 0.6 cm). Inflammatory markers in the blood were checked again after the conclusion of therapy, and they were normal. Further ultrasound of the abdomen and pelvis was performed 6 months later and showed normal liver architecture (no abscesses were present). 

## Discussion

*A. caviae* is the third most predominant strain of Aeromonas associated with human disease, with first being *A. hydrophila* and second being *A. sobria* [8]. Its ability to cause disease is based on several virulence factors identified, such as DNAase, hemolysin, heat-labile enterotoxin and serine proteases (also associated with the development of antibiotic resistance) [9]. The primary antibiotic regimen given in patients with *Aeromonas* spp. infection consists of fluoroquinolones, third and fourth generation cephalosporins and trimethoprim/sulfamethoxazole (TMP/SMX) [8]. However, the use of antibiotics, in combination with the expression of mobile resistance genes has led to the development of antimicrobial resistance [8]. To be more specific, the percentage of resistance to ceftriaxone and ciprofloxacin, for Aeromonas infection in the bloodstream, is estimated to be around 5–15% while in extrainstestinal infections, the percentage rises to 70.6% and 35.3% respectively [8]. In the case described, the patient was admitted due to fever, diarrhea and vomiting and upon imaging of the abdomen, liver abscesses were noted, while blood cultures returned positive for *A. caviae*. The patient’s past medical history is significant for pancreatic cancer (surgical resection) and lung cancer (lobectomy) which, according to the literature, predisposes to Aeromonas infection [6]. During hepatobiliary disease, increased intraductal pressure or stasis could explain the higher incidence of *A. caviae* infection in such patients [6]. Moreover, it is widely accepted, that chemotherapy breaks the intestinal mucosal barrier which, in combination with the toxic effect it has on neutrophil function, makes the host susceptible to opportunistic infections [6]. Consumption of contaminated fish can predispose to Aeromonas infection [6] and in the case described no specific dietary habits were mentioned, so exposure to fish is not known. The stool culture was negative for *Salmonella* spp., and *Shigella* spp., which can often co-exist with Aeromonas infection [10]. As mentioned above, the formation of abscesses can be a complication of *Aeromonas* spp. infection, with a few case reports describing hepatic abscess due to *A. sobria* and *A. hydrophila* [5]. Oladele et al. [11] reported the formation of micro-brain abscess in fish who suffered from *A. caviae* bacteremia. We conducted a literature search on Google scholar, PubMed and Scopus with the terms *A. caviae* and hepatic abscess, with no reports found. To our knowledge, this represents the first case of liver abscess due to *A. caviae* infection. 

## Conclusion

As stated previously, *Aeromonas* spp. is a Gram-negative facultative anaerobic, opportunistic pathogen that can infect people with underlined hepatobiliary disease, such as the patient presented [6]. *Aeromonas* spp. have been frequently detected in treated drinking water, often forming biofilms [12]. The latter poses a significant public health concern, as this can bring serious health risks to the consumers [12]. As mentioned above, antimicrobial resistance percentages are rising, with water being a possible reservoir of resistance genes [8], [12]. Finally, climate change can possibly disrupt aquatic ecosystems by increasing *Aeromonas* spp. bacterial growth and biofilm production which can have devastating effects on global human health [13]. 

## Notes

### Author’s ORCID

- Aristos A: https://orcid.org/0009-0003-6567-1999

### Ethical approval 

The patient was informed and gave his consent both verbally and in writing. 

### Funding

None. 

### Competing interests

The authors declare that they have no competing interests.
